# Facilitation of object encoding in infants by the observation of giving

**DOI:** 10.1038/s41598-021-97910-3

**Published:** 2021-09-15

**Authors:** Denis Tatone, Mikołaj Hernik, Gergely Csibra

**Affiliations:** 1 Department of Cognitive Science, Central European University, Budapest, 1051 Hungary; 2 Department of Psychology, UiT The Arctic University of Norway, 9019 Tromsø, Norway; 3 Department of Psychological Sciences, Birkbeck, University of London, London, WC1E 7HX UK

**Keywords:** Human behaviour, Anthropology

## Abstract

We propose that humans are prepared to interpret giving as a diagnostic cue of reciprocal–exchange relations from infancy. A prediction following from this hypothesis is that infants will represent the identity of an object they see being given, because this information is critical for evaluating potential future reciprocation. Across three looking-time experiments we tested whether the observation of a transfer action induces 12-month-olds to encode the identity of a single object handled by an agent. We found that infants encoded the object identity when the agent gave the object (Experiment 1), but not when she took it (Experiment 2), despite being able to represent the goal of both actions (Experiments 1 and 3). Consistent with our hypothesis, these results suggest that the infants’ representation of giving comprises information necessary for comparing the value of transferred goods across sharing episodes.

## Introduction

Humans regularly engage in the active transfer (i.e., giving) of goods among genetically unrelated individuals^1^. The centrality of giving across human societies is testament of the key role that sharing of resources played throughout our evolutionary history^2,3^. It has been argued that this behavior evolved in the context of so-called reciprocal–exchange relations, which are stable dyadic associations sustained through the asynchronous and reciprocal sharing of goods (often of the same kind)^4,5^. These relations provided a buffer against the risk of food shortages that characterized our ancestral foraging niche^6^. The stability of these relations however required reliable ways of signalling the commitment of the parties to the continuation of the exchange, as partners may grow stingy or find new group members to interact with^7,8^. Under this account, giving was recruited to support reciprocal–exchange relations because, owing to its costliness and selectivity, it is suitably designed to fulfill this signalling function. This is not the case for other transfer behaviors, such as tolerated taking, which leave the donor’s prosocial intent ambiguous^9,10^.

The recurrent co-variation of giving and reciprocal–exchange relations across our evolutionary history that this account presupposes has implications for social cognition. Multiple lines of evidence suggest that humans are sensitive to relationally informative cues: i.e., behaviors which, in virtue of their reliable occurrence within particular social contexts, can be used to diagnose the presence of specific social relations^11–13^. A giving action could act as such a cue by allowing people to infer the presence of a social relation between donor and recipient that is based on reciprocal exchange.

The preparedness to draw such an inference might emerge early in development. One-year-old infants have been shown to readily discriminate between giving and similar actions (such as tolerated taking), and to represent such an action in a schema that includes the specific agents involved in the observed interaction^14–17^. While this information is essential to track further interactions between donor and recipient, it is not sufficient to monitor whether an exchange is reciprocal and balanced. A giving action includes a host of information that can be used to monitor the state of the exchange across transfer episodes: e.g., the direction of transfer (who gave to whom), which allows detecting whether any reciprocation occurred; the number or amount of resources transferred (how much was given), which allows comparing the magnitude of benefits given and received; and the identity of the transferred resource (what was given), which allows assessing whether reciprocation is in-kind (and thus presumably matching the value of previous donations) or not. In the present study, we will focus on this third type of information. The proposal that infants might infer reciprocal–exchange relations from the observation of giving leads to the hypothesis that they should spontaneously encode the identity of the transferred object in the representation of giving events.

We tested this hypothesis across three looking-time experiments by investigating whether 12-month-olds would detect the change of an object transferred through a giving action, when the object is the only item in the scene. We showed infants events involving only one item because it is well established in the literature on early goal attribution that infants are insensitive to changes in object identity when the object acted upon is the only item available^18–22^. We reasoned that if the observation of giving facilitates the encoding of object identity, infants should register this information even if there is only one object to be acted upon (Experiment 1). To ascertain that object encoding was induced by giving rather than by other non-specific effects that this action resulted in (such as the object’s relocation and its proximity to an agent), we measured how infants responded to object changes in closely matched taking events (Experiment 2). Finally, to examine whether infants represented the goal of a taking action irrespective of object encoding, we tested whether infants detected a goal change after being exposed to taking events (Experiment 3).

Twelve-month-olds are an ideal age group to test the effects of giving on object encoding. On one side, infants at this age still fail to spontaneously encode the identity of singly acted-on objects^19^. On the other, they are already adept interpreters of giving events—even when these merely consist, much like the stimuli used in the current work, in the displacement of an object to a passive recipient^14,15^.

## General methods

**Figure 1:**
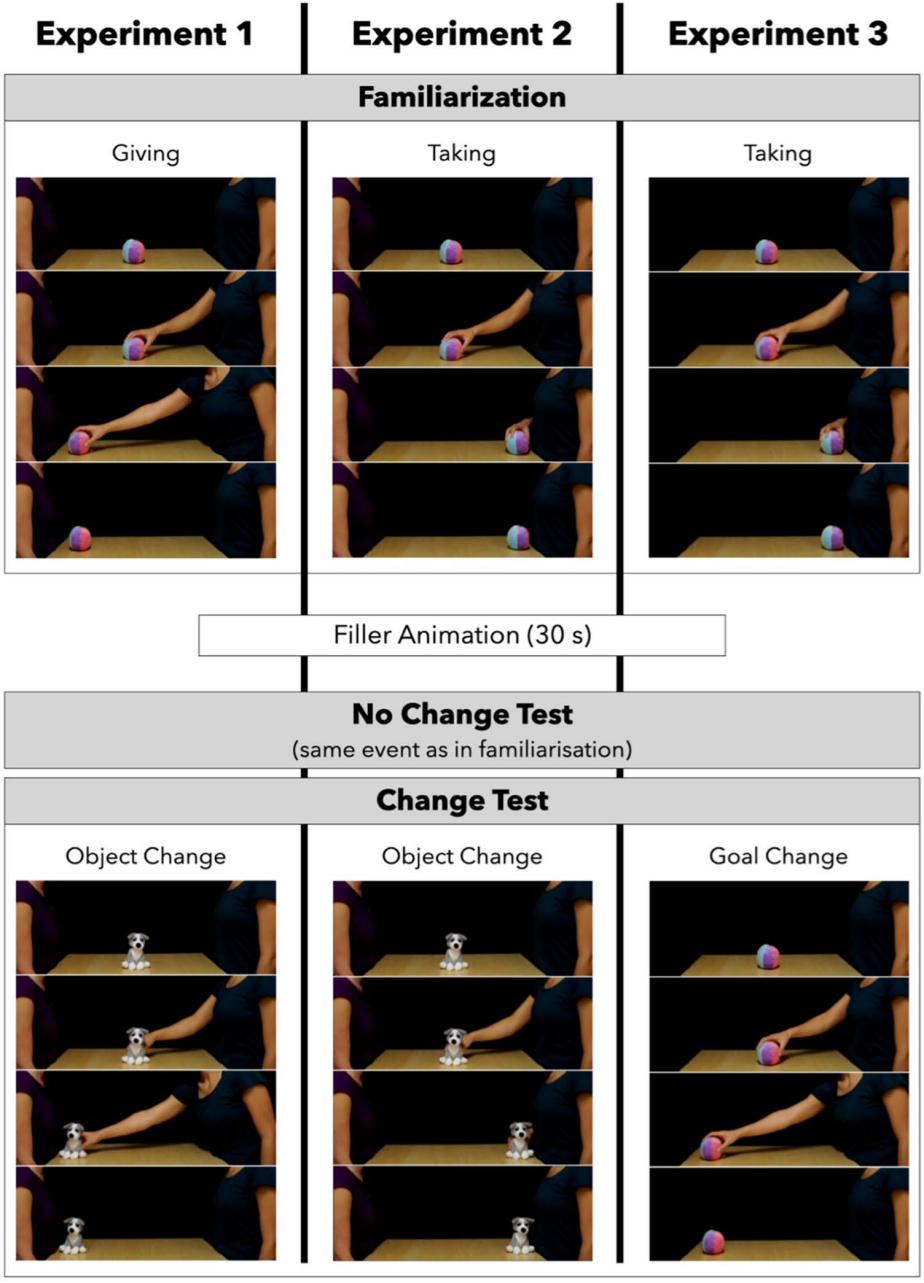
Schematic visualization of the events shown in Experiments 1–3.

All experiments had the same structure. Infants were shown a total of six events: four identical familiarization events followed by two test events (Fig. 1). The familiarization events consisted of short movies featuring an agent performing a giving (Experiment 1) or a taking (Experiments 2 and 3) action. After a filler animation (30 s), infants were presented at test with the event that they had seen in familiarization (No Change test) as well as with new event (Change test), where the agent produced either the same action on a new object (Experiments 1 and 2), or a new action on the same object as in the familiarization (Experiment 3).

We recorded infants’ looking times during and after the familiarization and test events. Our main measure of interest was the amount of looking allocated to the outcome of each test event. For further details on data collection and treatment, see Coding and Data Analysis.

## Experiment 1: object change (giving)

In Experiment 1 we tested whether the observation of giving actions would induce infants to encode the identity of the transferred object. We familiarized infants with agent A giving an object (e.g., a ball) to agent B, and later showed the same agent giving the same object or a new one (e.g., a plush dog) to B. If infants encoded the identity of the object, they should detect the object change, thus looking longer at the test event, which featured the new object.

### Results and discussion

**Figure 2:**
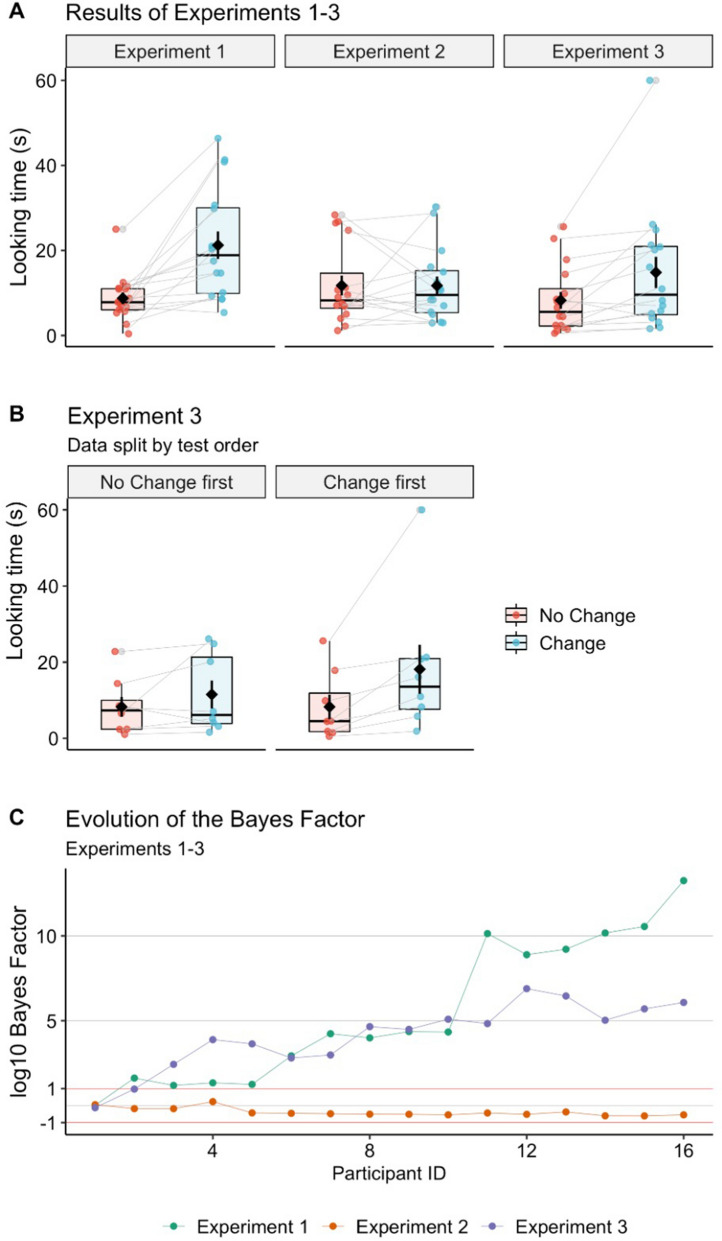
(**A**) Raw looking times in Experiments 1–3. (**B**) Raw looking times in Experiment 3 split by Test Order (No Change first vs. Change first). Black diamonds represent means and error bars represent ± 1 standard error of the mean. The bottom and the top of the boxes represent the first and the third quartiles. Whiskers extend from the middle quartiles to the smallest and largest values within 1.5 times the interquartile range. Dots connected across boxes represent raw looking times that each participant contributed across test events. (**C**) Evolution of log10-Bayes Factor over the course of data collection in Experiments 1–3. Values larger than + 1 indicate a strong effect in the predicted direction.

As predicted, infants looked longer to the Change test (*M* = 21.23 s, *SD* = 12.96 s) than to at the No Change test (*M* = 8.76 s, *SD* = 5.44 s) (Fig. 2A). This was confirmed by an ANOVA with Test Type (Change vs. No Change) as within-subject factor and Test Order (Change first vs. Change second) as between-subject factor, which revealed only a significant main effect of Test Type, *F*(1, 14) = 13.46, *p* = 0.003, η_*p*_^2^ = 0.49. Looking durations differed from each other also by Wilcoxon signed-ranks test (*p* = 0.001) and by Bayes factor comparison (log10-BF = 13.256), thus indicating strong evidence for H_1_ (Fig. 2C). The effect was visible also at the individual level, with 15/16 infants looking longer to the Change test.

The results show that infants reliably detected the object change, which suggests that the representation of the giving action included information about the identity of the transferred object.

## Experiment 2: object change (taking)

Lack of sensitivity to object change in single-item events has been reported in studies using simple grasping and reaching actions that, unlike the giving actions presented in Experiment 1, produced no conspicuous object displacement^19,22^. For this reason, it is possible that in Experiment 1, the relocation of the object, rather than the specific action performed on it, induced its encoding by increasing its salience. To control for this possibility, we presented a new group of infants with taking events closely matched for overall object displacement and tested whether they would similarly detect the change of object identity at test.

### Results and discussion

Unlike in Experiment 1, infants looked similarly to the No Change test (M = 11.75 s, SD = 9.29 s) and the Change test (M = 11.73 s, SD = 8.52 s) (Fig. 2A). An ANOVA conducted in the same manner as in Experiment 1 revealed no significant main effect, *F*(1, 14) = 0.11, *p* = 0.741, η_*p*_^2^ = 0.01, and no interaction, *F*(1, 14) = 0.743, *p* = 0.403, η_*p*_^2^ = 0.05. Non-parametric and Bayes-factor analyses yielded comparable results: *p* = 0.897 by Wilcoxon signed-ranks test; log10-BF = − 0.543 towards H_0_, indicating no difference (Fig. 2C). Eight of the 16 infants looked longer at the Change test event.

To compare infants’ looking behavior between the two experiments, we performed an ANOVA with Test Type (Change vs. No Change) as within-subject factor and Experiment (1 vs. 2) as between-subject factor. The analysis yielded a significant main effect of Test Type, *F*(1,30) = 9.35, *p* = 0.005, η_p_^2^ = 0.24, as well as a significant interaction, *F*(1,30) = 6.80, *p* = 0.014, η_p_^2^ = 0.19 (see Fig. 2A), which provides statistical support for the interpretation that looking-time patterns differed between Experiments 1 and 2.

The different reaction to the object change between the two experiments suggests that the type of action observed critically influenced the encoding of the object identity. Since both giving and taking caused a comparable amount of object displacement and culminated with the object in close proximity of an agent, these two factors alone could not account for the encoding of the object identity in Experiment 1.

## Experiment 3: goal change (taking)

While suggesting that the encoding of object identity was specifically induced by the observation of giving, the null results of Experiment 2 fall short of clarifying whether infants failed to encode the identity of the taken object or to represent the agent’s goal altogether (as suggested by some interpretations of the null results in the goal-attribution studies with single item^18,19^). The latter implies that infants, who can represent the goal of giving^14^, would at the same time fail to represent the goal of taking actions, despite these being familiar and structurally simpler actions requiring the integration of only two elements (an agent and an object) in the event structure. Such a failure would be puzzling, considering that 12-month-olds can readily distinguish the goals of giving and taking actions when familiarized to abstract events differing only in direction of transfer^14,15^. Instead, infants could have represented the taking action under a description that leaves the identity of the target object unspecified: “the agent takes *an* object”^23^. While still supporting goal attribution, such a description would not allow infants to detect changes of object identity.

To adjudicate between these two alternatives, in Experiment 3 we tested whether infants would react to a change of goal after being exposed to taking events. To this end, we familiarized a new group of infants to a taking action (as in Experiment 2), and later showed them the familiar action again (No Change test) as well as a new action (Change test) involving the same object, but a different goal (putting the object away). If infants represented the goal of the taking action, they should be able to detect the goal change, and thus look longer at the Change Test event.

### Results and discussion

As predicted, infants looked longer at the Change test (*M* = 14.83 s, *SD* = 14.75 s) over the No Change test (*M* = 8.27 s, *SD* = 7.93 s) (Fig. 2A). An ANOVA conducted in the same manner as in the previous experiments revealed a significant main effect of Test Type, *F*(1, 14) = 19.69, *p* = 0.001, η_*p*_^2^ = 0.58, as well as a significant interaction, *F*(1, 14) = 5.70, *p* = 0.032, η_*p*_^2^ = 0.29. Upon exploring the interaction, we found that infants looked significantly longer at the Change test when this was presented first, *t*(8) = 5.09, *p* = 0.001, *r*^2^ = 0.14, but not when it was presented second, *t*(8) = 1.38, *p* = 0.209, *r*^2^ = 0.03, thus suggesting that the infants’ overall tendency to look longer at the Change test was influenced by an additive order effect (Fig. 2B). Non-parametric and Bayes-factor analyses corroborated the main findings: *p* = 0.004 by Wilcoxon test; log10-BF = 6.077, indicating strong evidence in favor of H_1_ (Fig. 2C). The effect was visible also at the individual level, with 14/16 infants looking longer at the Change test event.

The results of Experiment 3 show that infants detected the goal change. This evidence suggests that the lack of sensitivity to the object change evidenced in Experiment 2, rather than being due to a failure to represent the goal of the observed action, reflected a goal description which did not include information about the identity or kind of the transferred object^23^.

It may be still argued that the longer looking at the Change test was merely induced by the change in motion-path of the agent’s arm rather than by the change of goal. We see two reasons to resist this interpretation. First, none of the studies on goal understanding that used actions lacking cues of goal-directedness as control conditions reported longer looking to path changes^18,19,24,25^ (despite being often presented as a complementary part of the picture on infant goal encoding, the alleged change of path effect has been found in only one published report^22^). Second, 12-month-olds have been shown to differentiate the goals of pushing-towards and pushing-away actions, which closely resemble the ones used in Experiment 3, even when these goals could only be inferred on the basis of minimal hand-grasp information^26^.

## General discussion

Across three looking-time experiments, we provided evidence that 12-month-old infants spontaneously include the identity of the transferred object in the representation of giving. Specifically, we showed that 12-month-olds detected the identity change of an object when transferred via giving (Experiment 1), but not when transferred via taking, despite the two actions being equated for spatial displacement and final proximity to an agent (Experiment 2). This contrast allowed us to rule out the possibility that non-specific action effects, such as the relocation of the object, might have induced the encoding of the object’s identity, irrespective of the type of transfer observed. Furthermore, we showed that infants were able to detect a goal change from taking to putting away (in Experiment 3). This suggests that the lack of reaction to the object change in Experiment 2 was not due to a failure to interpret the goal of taking, but rather due to having set up a goal description which, unlike in giving, did not specify what kind of object was transferred (“She took *an* object”).

The present results demonstrate that infants spontaneously encode a type of information (object identity) essential to monitor and compare the value of goods exchanged across separate interactions. Complementing previous reports of selective encoding of transfer direction (who transferred to whom) for giving but not for taking events^15^, the current findings suggest that infants may spontaneously register a suite of information relevant to track the individual contributions of individuals participating in a giving interaction. Albeit initial, such evidence is compatible with the suggestion that infants may be prepared to interpret giving as a cue of reciprocal–exchange relations.

The present study contributes to the growing literature on early naïve sociology by suggesting that infants can leverage relationally informative cues in the domain of resource control and distribution not only to infer dominance^27,28^ or communal relations^15^, but also mutually beneficial associations between peers. This suggests that the conceptual lexicon of relational models that infants are endowed with may be richer than previously assumed^12^.

Our results have also broader implications for the developmental literature on goal attribution. The infants’ failure to encode the object identity in Experiment 2 is largely congruent with numerous studies attesting lack of sensitivity to object change after exposure to an action directed at an object, which is the only item in the scene^18,21^. At the same time, however, the infants’ looking-time response to the goal change in Experiment 3 suggests that goal attribution may occur in the absence of object encoding. This evidence warrants caution against interpreting absence of object encoding as evidence of failure to represent goals. Such an account importantly overlooks the possibility that actions may be interpreted as goal-directed despite including underspecified object representations^23^^,^^30^. Furthermore, the finding that object encoding can occur for single-item actions (Experiment 1) indicates, alongside earlier reports^18,19^, that evidence of contrastive choice is but one contextual factor able to induce feature-rich object representations in infancy. In fact, we expect a plurality of action domains to highlight object identity for functionally different reasons in a way that would merit the cognitive resources required for its encoding. Giving is one such instance, because the identity of the object earmarks the value of a material donation potentially subject to later reciprocation. Ostensive referential acts are another functionally distinct example in which information about object kind is registered because taken to convey culturally relevant information^31–34^.

Three caveats are due here. First, since our test events featured a kind change (e.g., from ball to plush animal, or vice versa), we do not know whether the representation of object identity evinced in Experiment 1 was restricted to a narrowly defined object category (e.g., *that* ball) or a more broadly defined kind (e.g., any ball), as it appears to be the case for the representation of grasping one of two objects^35^.

Second, even though two agents were present during both giving and taking events in Experiments 1 and 2, their participation to the two events was asymmetric. In giving the passive agent could be assigned a meaningful thematic role as recipient of the transferred object. This was not the case in taking, where the passive agent merely acted as bystander while an object not in her possession was being acquired. This asymmetry leaves open the possibility that the encoding of object identity may not be induced specifically by giving, but by transfer-mediated interactions more broadly. Future experiments employing taking actions targeting other individuals’ possessions and hence amenable to be construed as social interactions should help us experimentally address this concern.

Third, although giving and taking were equated in the overall amount of object displacement, some perceptual differences between these two events potentially contributed to the differential encoding of the object. For instance, because giving featured two extended arm movements (one to grab the object, another to transfer it), whereas taking featured only one, the “extra” movement of the actor’s arm in the former event might have caused infants to pay more attention to the object acted upon.

It is worth noting that the giving stimuli used in this study featured completely passive agents, who did not communicate nor acknowledge receiving the object. Despite such skeletal implementation of giving, the evidence from Experiment 1 suggests that infants construed the event as a social interaction. This evidence corroborates prior findings with animated stimuli showing that minimal cues of possession transfer suffice to induce the interpretation of giving^36^ and extends this conclusion to stimuli featuring human actors.

To conclude, the hypothesis tested in the present study followed from the proposal that infants interpret giving as the episodic manifestation of reciprocal–exchange relations and consequently encode information relevant for monitoring the state of transactions within these relations. Here we showed that, by the time that infants interpret giving as an instance of social interaction between two specific agents^14^, resource identity is already integral part of their representation of giving. Discovering which other types of information (e.g., number of resources transferred) is integrated within this representation, and at which age, remains a question for future studies. Whichever ontogenetic trajectory the enrichment of the concept of giving may take, furnishing it with minimally distinctive information about the item transferred represents a crucial developmental step towards more elaborate forms of bookkeeping, which are essential to navigate social relations based on changing accounts of balance.

## Methods

All experiments shared same design structure. Infants were familiarized to four identical familiarization events and subsequently shown two test events: one, which was identical to the familiarization events (No Change test), and another, which featured an object or action change (Change test), depending on the experiment.

### Experiment 1

#### Participants

Sixteen infants participated in the experiment (6 females; mean age = 363 days; range = 356–370 days). Four additional infants were excluded due to not looking during the events (n = 3) and crying during the familiarization (n = 1).

#### Apparatus and procedure

The infants were tested in a dimly lit soundproofed room. They sat on the parent’s lap, about 100 cm away from the presentation screen (a 102 cm wide-screen LCD monitor set on a 1920 × 960 resolution). A hidden camera mounted under the screen recorded infants’ looking behavior at 25 frames per second. The parent was asked to close her eyes and to avoid interacting with the infant during the testing.

Each familiarization event was preceded by a short attention-getter: a shaking black checkerboard against grey background (1 s). At the end of the fourth familiarization, a filler animation consisting of a series of pulsating checkered tiles on a black background was played (30 s). All familiarization and test animations were accompanied by a continuous melodic tune.

#### Stimuli

The familiarization events started with two agents (A and B, wearing a purple and a dark green t-shirt, respectively) sitting at the opposite ends of a table, equidistantly from an object located at its center. The object was either a striped plush ball or a plush dog, both of which are familiar object kinds for 12-month-olds. The videos were edited so that only the torso, arms, and hands of the agents could be seen, but not their heads, in order to avoid that the infants may have reacted to subtle communicative or emotional cues inadvertently provided by the agents during the transfer.

The familiarization event (4 s total running time) consisted in the following sequence: agent A reached for the object, grasped it, lifted it, placed it on the opposite side of the table in front of agent B, and withdrew her hand under the table (Fig. 1). Agent B remained motionless throughout the transfer, never touching or grasping the object. The movie ended with the object placed in front of B (Fig. 1). Looking times were measured from the last frame of the movie, until the infant looked away for at least 2 s or 60 s elapsed.

The No Change test was identical to the familiarization videos, whereas the Change test event consisted in the same giving action transferring the other object. The two test events were matched for overall duration, timing, and action kinematics.

The identity (A vs. B) and side (left vs. right) of the agent transferring the object, as well as the identity of the object (ball vs. dog), and the order of test events (Change first vs. Change second) were fully counterbalanced across infants.

#### Coding and data analysis

We performed an offline frame-by-frame analysis of looking behavior. Looking times in familiarization and test were measured from the end of the action to when the infant looked away from more than 2 s or looked cumulatively for 60 s. To be included in the final data analysis, infants were required to watch each of the six events for the whole duration of the action (4 s). Infants who did not meet this criterion were labelled as “not looking during the events”. Half of the final sample was randomly selected and re-coded by a coder blinded to the hypotheses tested. The inter-coder agreement was excellent (Exp. 1: *r* = 0.990; Exp. 2: *r* = 0.963; Exp. 3: *r* = 0.965).

The looking-time data was log-transformed following the recommendations of Csibra and colleagues^37^. The transformed data was subjected to parametric analyses (repeated-measures ANOVA), whereas the untransformed data was subjected to non-parametric analyses (Wilcoxon signed-ranks test), which do not assume normality (an assumption that looking-times often fail to conform to). Additionally, we computed Bayes factors on log-transformed data (log10-BF) assuming variable effect sizes (after^37^). Bayesian analyses are especially relevant for Experiment 2, as they allow for concluding about the lack of effect. For ease of reading, we reported untransformed values in Fig. 2A, B, and log10-BF values in Fig. 2C.

### Experiment 2

#### Participants

Sixteen infants participated in the experiment (10 females; mean age = 362 days; range = 350–375 days). Four additional infants were excluded due to not looking during the events (n = 3), and caregiver interference (n = 1).

#### Stimuli

The familiarization and test movies featured the same agent and objects as those used in the previous experiment, but a different action (taking). Unlike in giving, after agent A grabbed and lifted the object, she placed it in front of herself, rather than in front of B. The duration, timing, and overall kinematics of the two transferring actions were otherwise identical.

### Experiment 3

#### Participants

Sixteen infants participated in the experiment (10 females; mean age = 364 days; range = 353–374 days). Five additional infants were excluded due to experimenter’s error (n = 2), not looking during the events (n = 2), and parental intervention (n = 1).

#### Stimuli

The movies used in the familiarization phase of Experiment 3 were the same as in Study 2, with the only difference that agent B was edited out from the scene. During test infants were presented with the taking action shown during familiarization (No Change test) and a new movie in which agent A placed the object to the opposite side of the table (Change test). The motion of this putting-away action was identical to that of the giving action used in Experiment 1, as it was directly obtained by editing out agent B from the event. Note that had agent B been present in the stimuli, the longer looking to the Change test would not provide unequivocal evidence for our hypothesis, because it could be driven either by infants detecting change of goal from taking to giving (if infants represented the goal of taking), or by infants perceiving for the first time a goal-directed action in the giving event (if infants did not represent the goal of taking).

### Ethical approval

The experiments employed only non-invasive procedures for assessing infants’ behavior. Infants were recruited through the Hungarian birth database. All parents were briefed about the nature and possible consequences of the study and signed an informed written consent before the study. All the experiments were approved by the United Ethical Review Committee for Research in Psychology (EPKEB) and conformed to the ethical rules and standards regarding psychological experimentation in Hungary.

## Data Availability

Data and stimuli can be found at the following OSF repository: https://osf.io/qvnfj/?view_only=e35a0bb6a83e4966b82a920dac477b82.
